# Development of a Data Logger for Capturing Human-Machine Interaction in Wheelchair Head-Foot Steering Sensor System in Dyskinetic Cerebral Palsy

**DOI:** 10.3390/s19245404

**Published:** 2019-12-07

**Authors:** Sotirios Gakopoulos, Ioana Gabriela Nica, Saranda Bekteshi, Jean-Marie Aerts, Elegast Monbaliu, Hans Hallez

**Affiliations:** 1 KU Leuven, Bruges Campus, Department of Computer Science, Mechatronics Research Group, Spoorwegstraat 12, 8200 Bruges, Belgium; hans.hallez@kuleuven.be; 2 KU Leuven, Department of Biosystems, Division of Animal and Human Health Engineering, Measure, Model and Manage Bioresponse (M3-BIORES), Kasteelpark Arenberg 30, 3001 Leuven, Belgium; ioanagabriela.nica@kuleuven.be (I.G.N.); jean-marie.aerts@kuleuven.be (J.-M.A.); 3 KU Leuven, Bruges Campus, Department of Rehabilitation Sciences, Research Group for Neurorehabilitation, Spoorwegstraat 12, 8200 Bruges, Belgium; saranda.bekteshi@kuleuven.be (S.B.); elegast.monbaliu@kuleuven.be (E.M.)

**Keywords:** data logging, human-wheelchair interaction, inertial measurement units, electric-powered wheelchair, time alignment, signal synchronization, dyskinetic cerebral palsy, dystonia, choreoathetosis, clinical tool

## Abstract

The use of data logging systems for capturing wheelchair and user behavior has increased rapidly over the past few years. Wheelchairs ensure more independent mobility and better quality of life for people with motor disabilities. Especially, for people with complex movement disorders, such as dyskinetic cerebral palsy (DCP) who lack the ability to walk or to handle objects, wheelchairs offer a means of integration into daily life. The mobility of DCP patients is based on a head-foot wheelchair steering system. In this work, a data logging system is proposed to capture data from human-wheelchair interaction for the head-foot steering system. Additionally, the data logger provides an interface to multiple Inertial Measurement Units (IMUs) placed on the body of the wheelchair user. The system provides accurate and real-time information from head-foot navigation system pressure sensors on the wheelchair during driving. This system was used as a tool to obtain further insights into wheelchair control and steering behavior of people diagnosed with DCP in comparison with a healthy subject.

## 1. Introduction

Nowadays, the number of people in the world who need a manual or powered wheelchair for their mobility is estimated at just over 65 million [1]. In the meantime, recent technological advancements have led to a decrease in the cost of electronic devices. As a result, an upsurge in the monitoring and recording of driving and physiological characteristics of wheelchair and wheelchair users respectively, has emerged. The monitoring methods employ a variety of electronic monitoring technologies, also known as data loggers [2].

As reported in [2], in the case of manually operated wheelchairs, half of the data loggers described in the literature are placed on the user, and the rest of them are installed on the wheelchair. Regarding the user, the majority of the data loggers use accelerometers and heart monitors, while on the wheelchair odometers and accelerometers are used. On manually operated wheelchairs, the most reported outcomes are distance, mobility events, heart rate, speed/velocity and acceleration. As far as data loggers for electric-powered wheelchairs are concerned, more than 90% of the data loggers are installed on the wheelchair, whereas only a mere 10% of them are placed on the user. The most frequently used technologies are accelerometers, pressure sensors or switches, odometers and global positioning systems (GPS). The majority of the reported outcomes are pressure relief activities, distance traveled, mobility events and acceleration [3]. Despite the fact that data loggers are being increasingly used, to our knowledge there is no recorded attempt in the literature for logging data from the interaction between users diagnosed with dyskinetic cerebral palsy (DCP) and their wheelchairs.

Currently, around 17 million people worldwide live with cerebral palsy (CP), with the majority of those being children and teens [4]. CP has a prevalence of 1.3–3.1 per 1000 live births [5]. DCP is characterized by abnormal postures or movements associated with impaired muscle tone regulation or movement coordination, comprising two major movement disorder patterns: dystonia and choreoathetosis [5]. DCP is known as the most disabling CP type [5,6,7]. In DCP, 80% of the patients lack the ability to walk or to handle objects [8,9]. Consequently, the vast majority cannot steer an electric-powered wheelchair using a conventional joystick. In DCP, head-foot movements are basal motor patterns and can be better controlled than fine motor movements. Therefore, clinical practice supports the use of a head-foot steering system for this target population [5].

**Figure 1:**
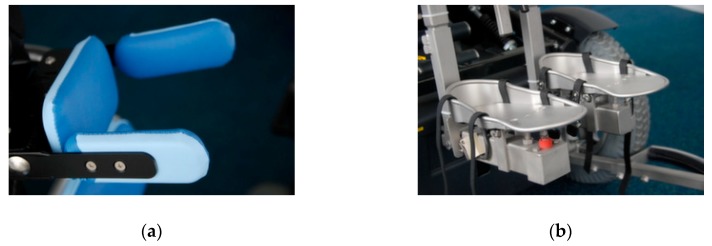
The Adremo head-foot wheelchair steering system used by the dyskinetic cerebral palsy (DCP) population. (**a**) Head support equipped with integrated mechanical switches responsible for steering right and left; (**b**) foot support equipped mechanical switches responsible for accelerating and braking.

Two systems may be used for the DCP target population: the “Permobil Head Array” (Permobil AB, Timra, Sweden) and the “Adremo Head/Foot System” (Adremo Revalidatietechiek B.V., Gorredijk, Netherlands). The “Permobil Head Array” is based on proximity sensors or mechanical switches installed on a head support and focuses on people with neuromuscular diseases and tetraplegic patients. However, this system is inappropriate for fast, large and strong involuntary movements, which are common in DCP patients. On the other hand, the “Adremo Head/Foot System” has shown good clinical results for the DCP target population. The “Adremo Head/Foot System” equips the wheelchairs with special head and foot supports (see Figure 1). By default, the function to accelerate and brake is done by foot, while steering is done using the head. The Adremo head-foot navigation system relies on pressure sensors, which are typically implemented in the head and foot supports as mechanical ON/OFF switches.

In order to get acquainted with and master the wheelchair control system, extensive drive training is mandatory for DCP patients. Consequently, there is a need for a clinical research tool that could increase the insights into wheelchair control and steering behavior of the user. The monitoring system would be a valuable tool as a continuation of the Dyskinesia Impairment Mobility Scale (DIMS) developed by the authors. The DIMS is a video-based tool that measures presence and severity of dystonia and choreoathetosis during powered mobility in DCP [10]. The aim of this paper is to propose a monitoring system, which can be easily integrated into the special head-foot wheelchair driving system that is used by DCP patients. The data logger captures the human-wheelchair interaction and provides an interface to multiple IMUs placed on the body of the wheelchair user.

This paper is organized into five sections. In Section 2, we go over the requirements, the development of the data logger and the experimental set up. In Section 3, experimental results, such as synchronization between the wheelchair data logger and the IMUs placed on the body of the user, power consumption and latency, are displayed. Furthermore, the signal acquisition capabilities of the system and an early comparison of driving characteristics between DCP patients and a health subject are presented. In Section 4, a discussion and our future plans are provided. Finally, Section 5 concludes the paper.

## 2. Materials and Methods

### 2.1. Wheelchair Head-Foot Steering System Function

Wheelchairs that are equipped with the Adremo system are controlled by the user via by mechanical, analog ON/OFF switches/sensors. The sensors that are installed on the wheelchair are responsible for controlling the following functions: gas pedal, electromagnetic brake enable/disable, steer right, steer left, select between forward and reverse, and enable/disable the driving system. An additional sensor on the left foot support can be assigned to further function. All sensor signals, apart from the gas pedal signal, are represented by 0 Volts (V) and 5 V, depending if each sensor is enabled or disabled. 0 Volts represent the “ON” state of the sensor, when the sensor is enabled. Accordingly, 5 V represent the “OFF” state of the sensor, when the sensor is disabled. However, a few wheelchairs do not follow the previously mentioned operational principle, as the commands “ON” and “OFF” are represented by 0 and 10 Volts respectively. The speed of the wheelchair is regulated via a linear motion potentiometer, which acts as a gas pedal.

### 2.2. Requirements of the Data Logging System for the Wheelchairs

Before the design of the wheelchair data logging system, its desired characteristics were taken into consideration. To begin with, a parameter of major importance is the portability of the data logger, as it should be small in size and battery powered. Moreover, the data logger should store the acquired data on a removable storage media, in order to ensure that data are easily accessible and transferable. Furthermore, the installation of the device should be quick and easy in less than five minutes. It should be ensured that clinicians and caregivers can install the data logger without any prior knowledge or technical support. What is more, the data logger should not affect the functionality of or cause interference or damage to any of the electrical parts of the wheelchair. Additionally, the data logger should be compatible with sensor signals from various pressure sensors which Adremo head-foot steering system uses. Finally, the data logger must be reprogrammable and reconfigurable, in order to provide the ability to extend its data log functionality based on the number and type of the wheelchair sensors.

### 2.3. Microcontroller Platform Selection

There are numerous platforms based on microcontrollers available on the market [11,12,13,14,15,16]. In this study, an Arduino Mega 256 R3 (Arduino, Italy) microcontroller development board was selected for numerous reasons. The use of Arduinos has many benefits, as a low development cost can be achieved. Moreover, Arduinos offer hardware simplicity and every implementation can easily be duplicated. Additionally, libraries for an extensive collection of modules are available, in order to interconnect the platform to any design and specification. Finally, their usability has been proved in the literature in plenty of scientific studies.

In wheelchairs, Arduinos have been mainly used for monitoring and/or logging physiological signals from the user. In [17], a posture recognition system based on pressure sensors installed on the seat of the wheelchair was proposed. In [18] the previously mentioned posture recognition system was extended, featuring activity recognition. Likewise, a pressure and temperature acquisition device was designed in [19] with the aim of preventing ulcer pressure. A more complex Arduino-based monitoring system was presented in [20], where force sensors, accelerometers, temperature sensors, and pulse sensors were integrated into a wheelchair. Finally, a wireless monitoring system was proposed in [21], capable of capturing wheelchair tilt, activity level, heart rate, temperature and humidity.

### 2.4. Data Logger Development

#### 2.4.1. Data Logger Hardware

The data logger itself consists of the following main components: a microcontroller (MCU) board, a data logger shield with a micro SD card adaptor shield and real-time clock (RTC), a Bluetooth (BT) module, and a custom-made data acquisition board. The MCU board is based on the low-power microchip 8-bit AVR ATmega2560, which has 16 MHz clock speed and 256 kilobytes (KB) of flash memory. The data logger SD card adaptor shield [22] can save data up to 32 gigabytes (GB) on any FAT16 or FAT32 formatted SD card. The data logger shield also incorporates a 3.3 volts (V) level shifter circuitry that prevents damage to the SD card. The SD memory card communicates with the MCU via serial peripheral interface (SPI) input/output pins. Four digital pins are used for SPI communication with the SD card: (1) as a chip select (CS) line to avoid communication conflicts with other SPI devices, (2) master-out-slave-in (MOSI), (3) master-in-slave-out (MISO) and (4) the bus clock line (SCL). A high accuracy RTC is embedded in the logger shield, and the RTC is attached to the MCU via an inter-integrated circuit (I2C) interface. The RTC is responsible for timestamping all the data with Unix timestamps and also real time. The BT module [23] is connected to the MCU using universal asynchronous receiver-transmitter (UART) communication. The BT connection is responsible for transmitting data packets to a personal computer for real-time visualization of the wheelchair head-foot steering system control commands. For the real-time data visualization, Java telemetry viewer software was utilized [24].

In order to acquire signals from the sensors of the wheelchair, a data acquisition circuit board was designed and developed. The current of the wheelchair sensors is 200 milliamperes (mA), while the voltage ranges between 0 V and 10 V. Consequently, there is no compatibility with the maximum input current and voltage of the MCU. The purpose of the data acquisition board is to convert voltage and current levels of the wheelchair sensors into MCU compatible levels. Based on the previous considerations, the additional data acquisition circuit was designed to comply with the specifications of the wheelchair and the MCU.

In detail, hexadecimal (hex) inverters were used in order to assign “OFF” state to 0 V and “ON” state to 5 V. In wheelchairs, where the “OFF” state was represented by 10 volts, voltage dividers were used to convert 10 V into 5 V. Then, a hex inverter was used to assign “OFF” state to 0 V and “ON” state to 5 V. Current limiting resistors were employed before the hex inverter stage so as to limit the current from the sensors of the wheelchair within the analog and digital input limits of the MCU. In cases where signal conditioning is not needed, signals were routed to the microcontroller only via current limiting resistors. Finally, signal from linear potentiometers were routed to the analog inputs of the MCU via voltage dividers.

The digital inputs of the MCU are responsible for capturing the ON/OFF sensor signals. On the other hand, analog inputs with a 10-bit analog-to-digital converter (ADC) are responsible for capturing the analog continuous values of the linear motion potentiometers. Additionally, a 3-axis accelerometer was connected to the wheelchair data logging device. This accelerometer is used for the synchronization of the wheelchair data logger with the IMUs placed on the body of the user. The synchronization process and operation are discussed in Section 2.6.1. The data logger system is connected between the wheelchair sensors and wheelchair control electronics. The data are logged into the SD card and transmitted via BT for real-time visualization.

**Figure 2:**
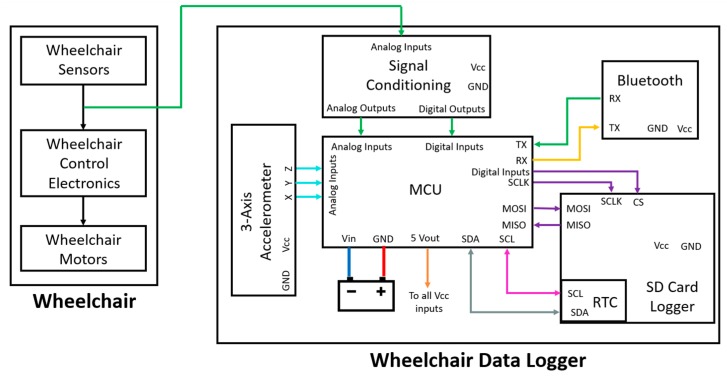
Connection diagram of the developed data logging device. The signal conditioning board is connected between the wheelchair sensors and the wheelchair control electronics. After the input conversion, data are fed to the microcontroller for logging and transmission.

**Figure 3:**
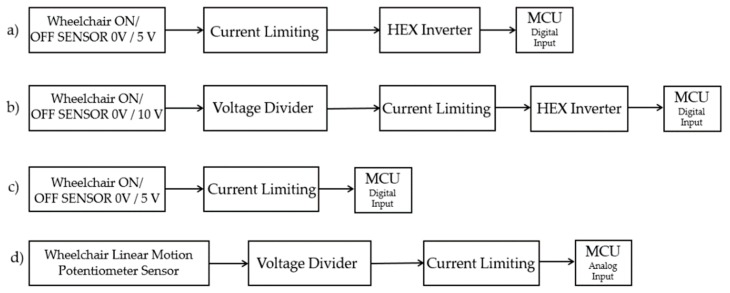
The schematic of the developed data acquisition board that shows the different data acquisition stages. (**a**) Depicts the stage where current limiting resistors and hex inverters are used; (**b**) shows the stage that consists of voltage dividers, current limiting resistors and hex inverters; (**c**) the stage that is used only when current limiting is needed; (**d**) the appropriate stage for capturing data from linear position potentiometer sensor of the wheelchair.

**Figure 4:**
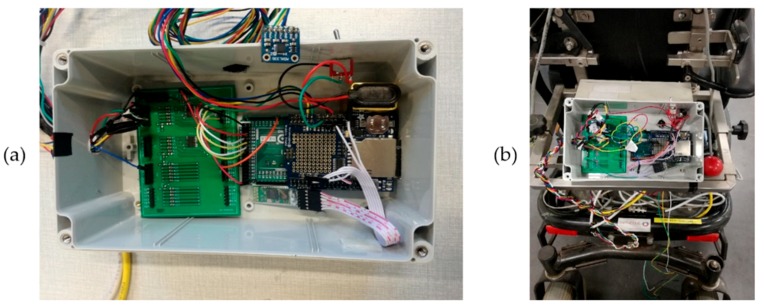
(**a**) The developed data logging system while testing in lab environment. (**b**) The data logging system installed on the wheelchair.

A diagram of the developed data logging system can be seen in Figure 2, while a schematic of the developed signal conditioning board can be seen in Figure 3. Figure 4 presents the actual data logging system in a lab environment and also mounted on the wheelchair. The dimensions of the wheelchair data logging device are 19 × 12 × 8 centimeters (cm) (length × width × height). The compatibility of the developed data logging device is guaranteed and tested in the wheelchair models presented in Table A1, Appendix A.

#### 2.4.2. Data Logger Software

**Figure 5:**
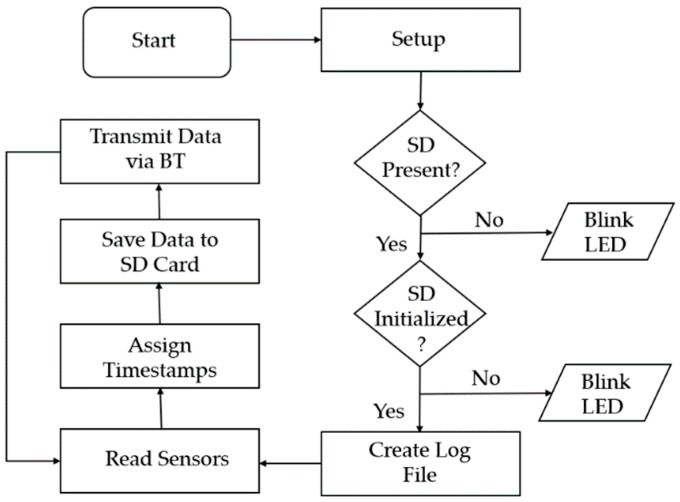
Flow diagram of the algorithm for the developed wheelchair data logging system.

For the data log software, initially, the required SPI, RTC, I2C and SD libraries are loaded. In the next step, the initialization of the analog and digital inputs, the SD card, the RTC and the Bluetooth takes place. An error check is performed to verify whether the SD card is inserted in the slot and if a new empty log file is created on the SD card. In case the SD card is not inserted in the slot or the log file cannot be created, a red light-emitting diode (LED) on the system flashes as an indication that an error has occurred. Upon the completion of the startup tasks, the program enters a repeating sample -store-transmit loop. The microcontroller reads the sensors of the wheelchair and the values of the accelerometer, then the raw values of the accelerometer are converted into acceleration units (m/s^2^). Following this, the data are assigned with a timestamp and fill in a buffer. This buffer is then flushed and saved on the SD card and transmitted via Bluetooth in real-time to the researcher’s personal computer for visualization. The real-time visualization also serves as a diagnostic tool to provide feedback in case of connection or data acquisition problems. The flow diagram of the developed algorithm is depicted in Figure 5.

### 2.5. 9-DOF IMUs Operation

**Figure 6:**
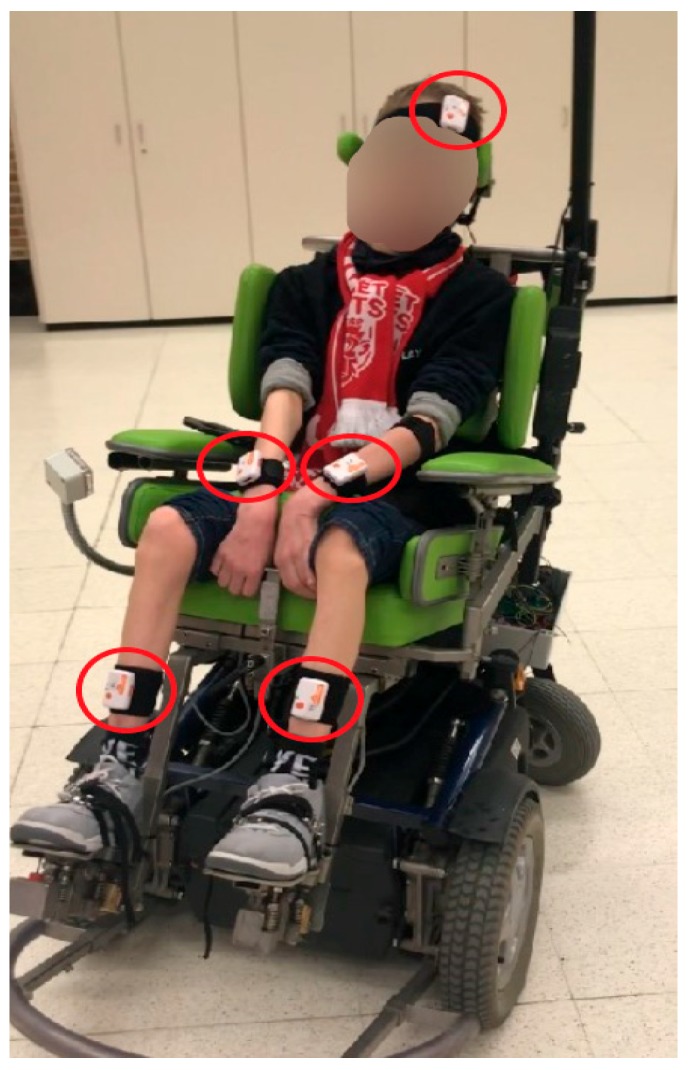
The five IMUs placed on a DCP patient while driving the head-foot wheelchair steering system. The IMUs are positioned on the right and left distal arm, on the right and left tibialis anterior, and on the forehead, respectively.

For monitoring upper limbs, lower limbs and head of the user and the movements of the wheelchair, a Shimmer 3 Consensys IMU development kit (Shimmer Sensing, Dublin, Ireland) was utilized. Five IMUs were placed on the right and left distal arm, right and left tibialis anterior, and forehead of the user, respectively, as shown in Figure 6. Additionally, an IMU was mounted on the wheelchair itself. The function of IMUs is to log acceleration and gyro events from the user’s body and also to keep themselves synchronized in time with each other. For this purpose, the IMU mounted on the wheelchair is assigned as the “master” device, while the IMUs placed on the body are assigned as “slaves”. The master IMU establishes a BT communication with the slave IMUs. The master IMU holds a list of the slaves’ media access control (MAC) addresses and generates the synchronization queue from the list of all slave IMUs. For each successful communication between the master and the slaves, multiple timestamps are shared with a resolution of 64 bits. Slave IMUs receive the timestamp of the master and record the time on their own clock. This process is repeated multiple times until the slave IMUs have a sufficient amount of sample sets. This way, the offset between the clocks of the slaves and the clock of the master can accurately be estimated, and the error due to the BT latency is minimized. Consequently, samples from all IMUs are logged under a common reference clock. The above discussed process is embedded into the Shimmer Consensys firmware.

### 2.6. Expermental Set up, Device Characterization

#### 2.6.1. Signal Synchronization of Wheelchair IMU Data and Wheelchair Sensors Data

Due to the fact that two independent data logging systems were utilized, the signals on each device were sampled asynchronously. In order to cooperatively use the acquired data, data from the two measurement systems need to be aligned in time (i.e., synchronized), in the view of the fact that a post-processing analysis should be conducted. For this reason, Matlab’s (Mathworks, MA, USA) cross-correlation function was employed.

Cross-correlation measures the similarity between two signals as one of the signals is shifted with a time lag. Cross-correlation to estimate the time delay between two frequently sampled signals, with the aim of time alignment between them, has long been utilized [25]. In the literature, cross-correlation has been implemented for signal synchronization in pattern recognition in driving behavior and the design of advanced driver assistance systems [26]. Moreover, cross-correlation has also found application in neurophysiology and biomedicine for synchronizing electrocardiogram (ECG) signals [27,28]. In addition, the utilization of cross-correlation for monitoring sudden events, such as earthquakes, in civil infrastructures has been reported in [29], while in [30], time align cross-correlation was employed for synchronizing GPS and IMU data of a remote-controlled aircraft.

In this study, the data logging system that captures the wheelchair’s sensor events has a sampling frequency of $f_{n}$ = 13.735 Hz, while the IMUs on the users’ body have a sampling frequency of $f_{m}$ = 51.28 Hz. Since data from the data logger share the same clock and data from the IMUs share another common clock, the method to synchronize signals from the two logging systems is to record the same signal with each data acquisition system. For this purpose, the data logger’s 3-axis accelerometer is placed on exactly the same spot as the master IMU. Thus, both accelerometers capture the same event. The signal synchronization is performed off-line after the data collection. Initially, due to the fact that accelerometer data are noisy, a low-pass filter with a cut-off frequency $f_{c}$ = 6 Hz is applied to data logger’s accelerometer and the master IMU’s accelerometer. Afterwards, the signal coming from the device with the highest sampling frequency is resampled, and the data are linearly interpolated by a factor p and then decimated by a factor q. To verify that this produces the desired rate, Equation (1) is used:

(1) $$f_{n}=\;f_{m}\;\left(p/q\right).$$

In the cross-correlation procedure, *m*(*t*) is the data logger’s accelerometer and *n*(*t*) is the master IMU’s accelerometer waveforms over interval T. The cross-correlation function is given by Equation (2):

(2) $$R_{nm}\left(\tau \right)=\;\overset{T}{\underset{0}{\int }}n\left(t\right)m\left(t+\tau \right)dt\;,$$

where $R_{nm}$ is the averaged product of m(t) lagged with respect to n(t).

After the analysis of the cross-correlation, the index of the highest peak is found and the difference between the two signals (lag) in seconds is calculated. When the value of cross-correlation is high for some value of the lag, with the ideal cross-correlation value being 1, it can be stated that n(t) and m(t) are similar at this lag value. Next, the lag value is applied to the wheelchair’s data logger timestamps and consequently adjusts them, so as the time alignment be achieved. Finally, the combined and synchronized data sets of the two devices are saved for future processing.

#### 2.6.2. Power and Current Consumption Measurement

Power consumption estimation is a typical step in the development of battery-powered, microcontroller-based equipment. The most common approach for power consumption measurement is the use of a shunt resistor for current sensing. In this method, a current shunt resistor is placed either in the power supply line (high side current sensing), or in the ground line (low side current sensing) [31]. The method is based on measuring voltage drop across the current shunt resistor that ranges between tens and hundreds *mΩ* with a tolerance no higher than 1% [31,32].

Before selecting the appropriate current sensing circuit for this specific application, it must be selected whether the current sensing will take place in the high side, or in the low side of the data logger. Low-side sensing causes a slight increase in the load’s ground voltage, consequently, the load’s ground is floating on higher voltage than the system ground. Moreover, low side sensing is suitable for measuring large currents and isolated loads [33]. On the other hand, high side sensing reacts quicker to changes in the current flow, and it does not cause disturbances to the ground of the system. However, high-side sensing requires the precise matching of the proper differential amplifier [33]. Thus, high side sensing was selected, by utilizing the INA219 Current Sensor Breakout. INA has a sensing range between 0 and 26 V, and reports current, voltage and power with an accuracy of 0.5%, and in a 12-bit resolution [34].

**Figure A1:**
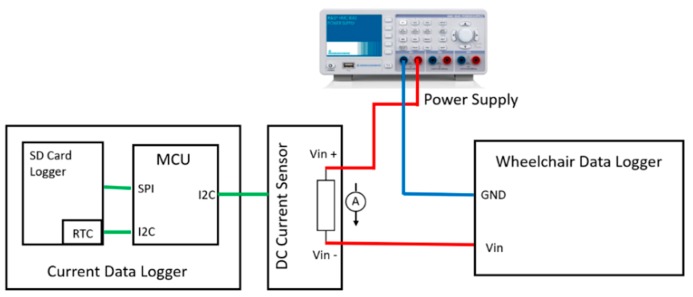
The experimental set-up used for estimating the power and current consumption of the developed wheelchair data logger.

In order to estimate the power and current consumption of the data logger, the current sensor was connected to an Arduino Mega 2560 via I2C communication protocol. The voltage drop across the 100 mΩ resistor of the current sensor was recorded and logged to an SD card logger shield in a similar way to the wheelchair’s data logger configuration, as explained in Section 2.4.1 The experimental set-up that was used to estimate the power and current consumption is presented in Figure A1, Appendix B.

#### 2.6.3. Bluetooth Transmission Latency Measurement

As noted in Section 2.4.1, the wheelchair data logger is capable of transmitting the wheelchair control commands over Bluetooth to a personal computer for the purpose of visualization, while the user is driving the wheelchair. In packet-based protocols, such as Bluetooth, transmission latency is inherent. The implementation of the data logger and the Bluetooth transfer protocol result in the transmission of a large amount of data in one direction. Before estimating the transmission latency, a definition for latency must be given. In the wheelchair data logger, latency is defined as the time that it takes for a full data packet that contains all the wheelchair sensor data to get from the data logger to another device via BT.

**Figure A2:**
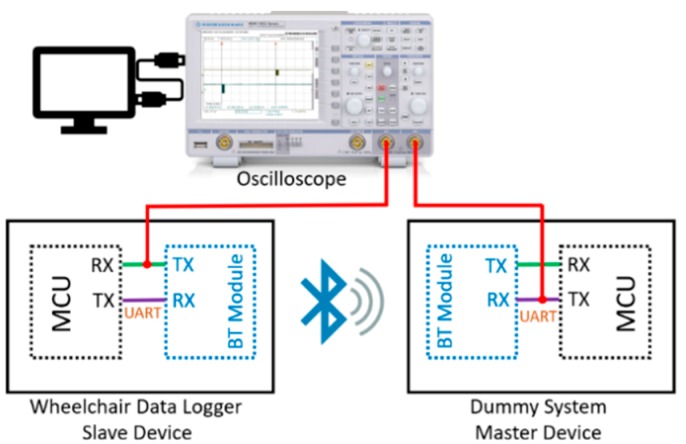
The experimental set-up used for estimating the Bluetooth latency.

The test setup for estimating the transmission over Bluetooth latency, consists of the wheelchair data logger and a “dummy” microcontroller-based device equipped with Bluetooth. The wheelchair data logger is configured as “slave” device and it has its normal functionality. The “dummy” microcontroller device is built using an Arduino Mega 2560, with an HC-05 BT module, and is configured as “master” device. The wheelchair data logger sends full data packets with the wheelchair sensors, and the “dummy” device receives them. During data transmission, one channel from an oscilloscope is used to capture data on the transmit pin (TX) of the wheelchair data logger UART communication. Another channel from the oscilloscope is used to capture data on the receive pin (RX) of the dummy data logger UART communication. Consequently, capturing the time interval between transmit and receive, the measurement of transmission latency is possible. The experimental setup used for estimating the Bluetooth latency can be seen in Figure A2, Appendix B. The oscilloscope is also connected to a personal computer in order for the screen of the oscilloscope to be captured. For this purpose, HMExplorer software (Rohde & Schwarz, Munich, Germany) was employed.

### 2.7. Implementation of the Wheelchair Data Logger in DCP Patient Group

Although this is not the focus of the paper, the data logging system was used to measure wheelchair movements and user movements in 10 DCP patients, while driving their electric-powered wheelchairs equipped with the Adremo head-foot steering system. Therefore, 10 patients diagnosed with DCP were selected from two special educational schools for people with motor disabilities. The participants were asked to perform various wheelchair driving tasks. The wheelchair data logging device was installed on the wheelchair. As previously mentioned, in order to log the user’s body movements, five IMUs were placed on the user’s right and left distal arm, right and left tibialis anterior, and forehead. An additional master IMU was mounted on the wheelchair itself. The accelerometer of the wheelchair data was placed on exactly the same spot as the master IMU, which was mounted on the wheelchair, for the purpose of synchronization between the two systems. Ethical approval was obtained from the Medical Ethics Committee UZ/KU Leuven, and informed assent and consent forms were signed by the participants and/or their parents. As results from this study have a scope which is more suited for a clinical journal, they will be published in a suitable journal.

## 3. Results

### 3.1. Electrical Proporerties

**Figure 7:**
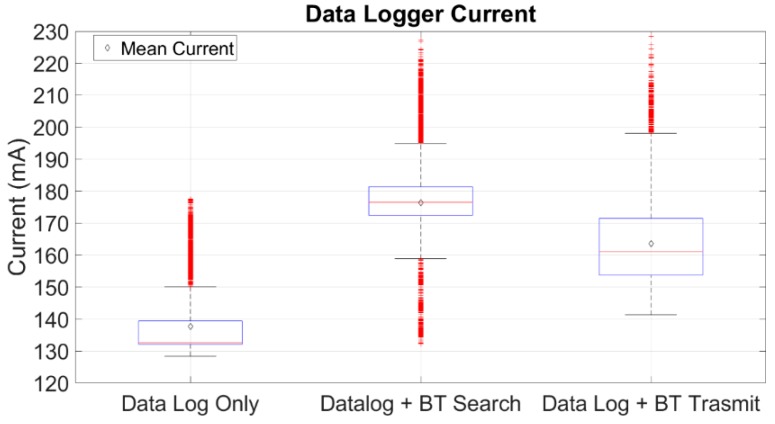
Current drawn by the data logger in three different states. Namely, when the device is performing only data log, when the device logs data and at the same time is searching for a Bluetooth device to be connected, and finally, when the device logs data and transmits data via Bluetooth to another device.

The current drawn by the wheelchair data logger and its power consumption were measured while the data logger was in three different operational states. The first operational state is when the data logger performs only data logging. The second operational state is when the data logger logs data and at the same time is scanning for a Bluetooth device in order to be connected. The third state is when the data are logged and are also transmitted via Bluetooth for visualization. It was found that the data logger draws a mean (μ) current equal to 137.64 mA, standard deviation (s) = 10.7552, when it operates only as a data logger. In the second operational state, when data logging is performed and the device is scanning for a Bluetooth device to be connected, the data logger draws a mean current equal to 176.44 mA, s = 16.0089. In the third state, when a Bluetooth connection with an external device is established, and the data are logged and transmitted, the mean current drawn by the data logger is 163.58 mA, s = 14.8273. The data logger’s current drawn results can be seen in Figure 7.

**Figure 8:**
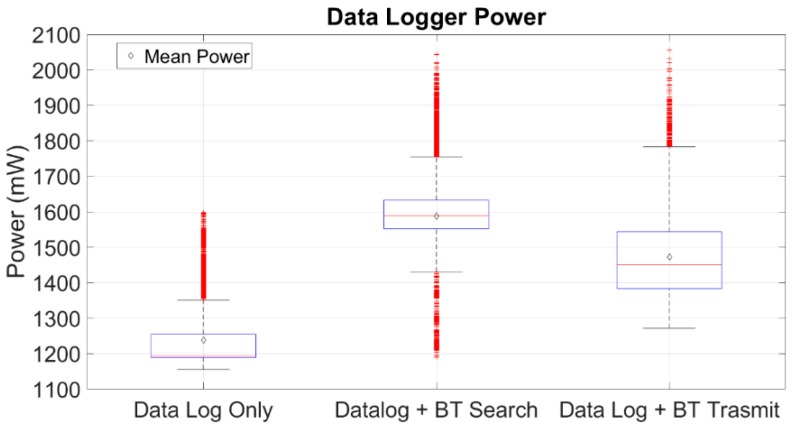
Power consumption of the data logger in three different states. Namely, when the device is performing only data log, when the device logs data and at the same time is searching for a Bluetooth device to be connected, and finally, when the device logs data and transmits data via Bluetooth to another device.

As far as the data logger’s power consumption is concerned, it was found that the data logger has a mean power consumption equal to 1238.76 mW, s = 96.7966, when it operates solely as a data logger. In the second operational state, when data logging is performed and the device is scanning for a Bluetooth device to be connected, the data logger has a mean power consumption of 1587.98 mW, s = 144.0802. Finally, in the third state, when a Bluetooth connection with an external device is established, and the data are logged and transmitted, the mean power consumption of the data logger is 1472.29 mW, s = 133.4455. The data logger’s power consumption results can be seen in Figure 8.

It is clearly visible that during the search process the Bluetooth modules consume more power. Having established a connection, the wheelchair sensors’ data are transmitted, and there is a considerable decrease in the power consumption. Furthermore, the instability of the power consumption is caused by two tasks. The first task is when the data are flushed to the SD data logger shield and they are logged onto the SD card. The second task is when the wheelchair sensors’ data packet is transmitted over Bluetooth. When the execution of these tasks takes place, there is a need for more current for the SD data log shield and the BT module. The data logger consumes more power for the logging of the data onto the SD memory card. It can be observed that when the data transmission over BT is active, the power consumption is increased by approximately 250 mW, or just by 19% in comparison with the state when only data logging is executed.

**Figure 9:**
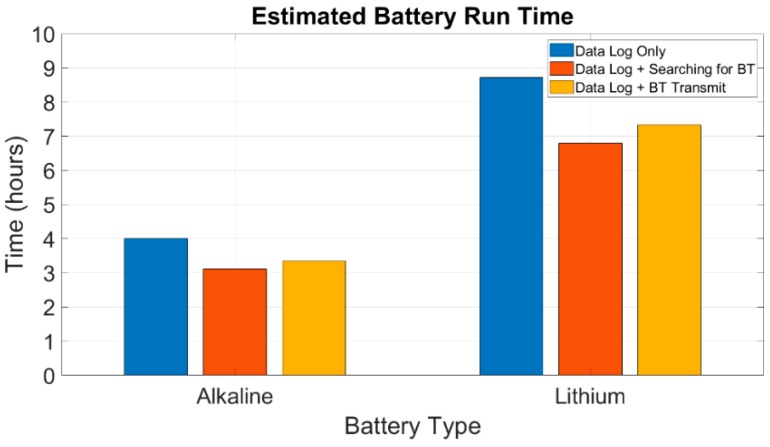
Estimated run time of the data logger when it is powered by alkaline or lithium 9V batteries, during three different operational states.

Since the data logger device can run on a common 9 V disposable battery, the run time of the data logging system was estimated when using two different types of commercially available batteries. The run time was estimated for alkaline and lithium batteries. The estimated run time can be seen as a bar graph in Figure 9. Given the fact that 9 V alkaline batteries have a capacity of approximately 550 mAh, when only data logging is performed, the device can run for about 4 h. When the device is searching for a BT device, while it is logging data, the run time drops down to 3.12 h. Provided that the device is logging data and transmitting the data via Bluetooth, the estimated run time was found to be 3.36 h. On the other hand, when lithium batteries, which have a capacity around 1200 mAh, are used to power up the data logger, in data log only mode, the device can be run for 8.72 h. Moreover, when the device is searching for a BT device, while it is logging data, the run time is 6.8 h. Additionally, when the device is logging data and transmitting the data via Bluetooth, the run time is 7.34 h. The device might have a high consumption, but it is still within our specifications, since it was used for monitoring driving tasks, whose duration was less than 30 min in total.

### 3.2. Bluetooth Transmission Latency

**Figure 10:**
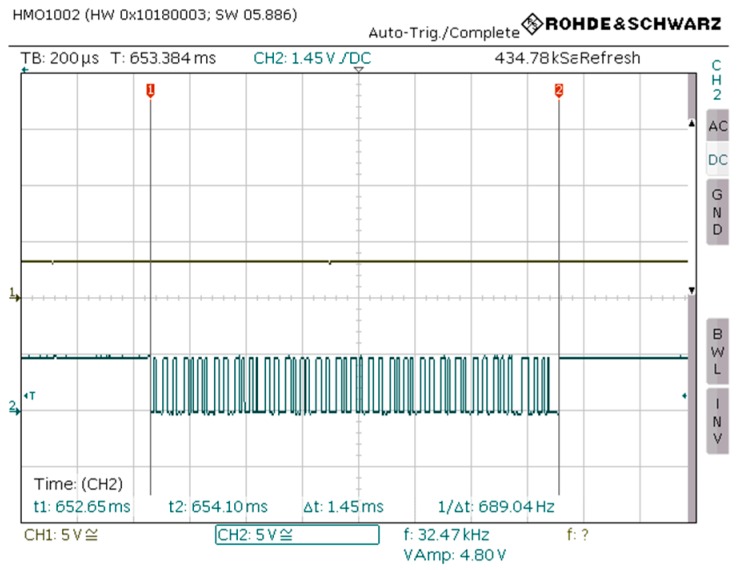
Time needed for the acquired wheelchair data to flow from the microcontroller through the Bluetooth module via UART interface.

Once a Bluetooth connection is established, acquired data from the data logger flows through the Bluetooth module via the UART interface. Then, the application inside the module bridges it to the Radio Frequency (RF) interface in order to be transmitted over the air. Since the MCU of the data logger is physically connected to the Bluetooth module via the UART interface, the time needed for the data to flow to the Bluetooth module can be estimated. The flow time of the data is defined solely by the UART baud rate that has been selected. In the wheelchair data logger, the baud rate is 38,400 bits per second (bps). The acquired data come from seven different wheelchair sensors, which are responsible for the following: gas pedal, electromagnetic brake, steer right, steer left, select between forward and reverse, enable/disable driving system, and an additional sensor on the wheelchair’s left foot support, which can be assigned for any other functionality. Each sensor data has a length of 8 bits, consequently, the total amount of data that has to flow through the Bluetooth module is 56 bits long. The time needed for this process can be calculated as 56/baud rate seconds and gives a result of approximately 0.001458 seconds, or 1.458 milliseconds. The experimental result on the actual data logger can be seen in Figure 10 and proves the above theoretical calculation.

**Figure 11:**
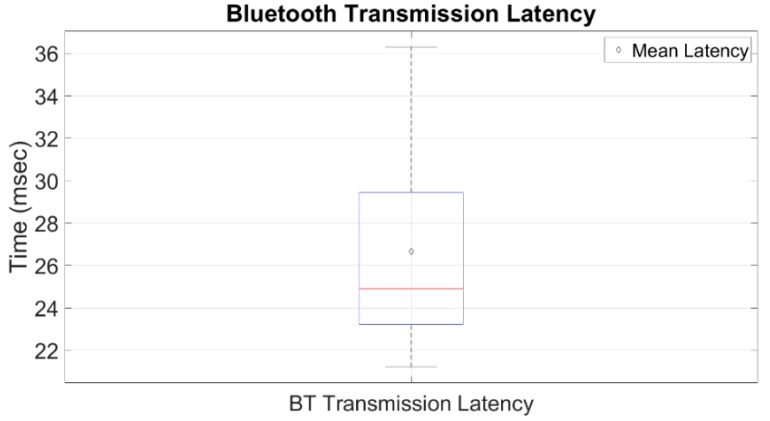
The Bluetooth transmission latency for a full packet that contains all data from wheelchair sensors.

Furthermore, as previously mentioned, transmission latency is defined as the time that it takes for a full data packet that contains all the wheelchair sensors’ data to get from the data logger to another device via BT. Since the transmission latency is not always constant, we repeated the data packet transmission 10 thousand times, so as to quantify the mean latency. The mean transmission latency was found to be 26.66 ms. The results can be seen in Figure 11.

Possible data packet losses during the BT transmission were also investigated. The BT module utilized for the data logger communicates with the MCU using UART protocol, as previously mentioned. The profile that the BT module adopts is a serial port profile (SPP) and is used to emulate a serial port connection over a BT device. SPP is ideal for transmitting bursts of data between two devices, and it is one of the more fundamental BT profiles. Using SPP, each connected device can transmit and receive data exactly as if there were RX and TX lines connected between them. Streaming packets that use SPP are lost when they are flushed. The flush function is when a packet enters the TX buffer of the controller [35]. Thus, if a packet enters the TX buffer it is guaranteed that it is not lost. However, despite the fact that a packet will not be lost in the TX buffer, it is possible that it might encounter a bit flip when it is transmitted over the air.

For this purpose, we examined both the packet loss and the corrupted data that could possibly be received. We flushed 1 million data packets (approximately 21 h of continuous data transfer) to the TX buffer of the data logger and transmitted them to the dummy BT device. On the dummy BT device, we measured the number of received packets, which consequently means the packets that were flushed to the TX line of the data logger. For every packet that the dummy device received, possible corrupted data were monitored by comparing the received data packet with the transmitted data packet. The distance between the two devices was set at five meters, without any obstacles between the devices, and neither packet losses nor corrupted data packets were encountered.

The dummy device successfully received 1 million packets. This could be explained due to the fact that a slow baud rate (38,400) was selected between the MCU and the BT UART interface. Moreover, data packets were relatively small (56 bits), and there was also enough time for them to be flushed into the Bluetooth’s TX line. Similar results were yielded in [36], where UART interface was used for steaming music on a BT connected headset and a high baud rate was set. Additionally, no bit flips occurred during the transmission; however, this might not apply in environments where electromagnetic interferences or heavy radiation are present [37].

### 3.3. Signal Synchronization Results

**Figure 12:**
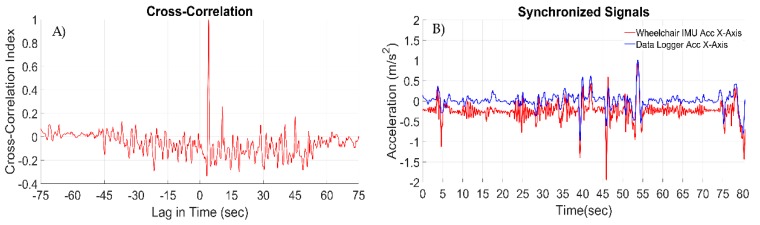
(**A**) The achieved cross- correlation index and the lag in time between the accelerometer of the wheelchair data logger and the accelerometer of the master IMU mounted on the same location on the wheelchair. (**B**) Synchronized accelerometer signals of the wheelchair data logger and the master IMU, both mounted on the same place on the wheelchair.

For the synchronization of the two independent data logging systems, a cross-correlation index = 1 was achieved. It should be noted that when the cross-correlation index = 1, this does not imply that both acceleration signals are exactly the same. The applied cross-correlation finds the similarities that occur in peak values of the signals and the synchronization is based on them. The achieved cross-correlation index and the lag between the two logging systems is depicted in Figure 12A. For the dataset that is presented in this paper, it can be observed that the lag between the two independent logging systems is 4641.5 milliseconds. The synchronized accelerometer signals from the two independent data logging systems are presented in Figure 12B. The accelerometer of the wheelchair data logger is visualized in blue color, while the one mounted on the wheelchair master IMU accelerometer is visualized in red color.

Since in our application the high-accuracy synchronization is not the requirement, the approach that was followed has proven to be acceptable. The data sets that are synchronized are short in duration (between one and seven minutes long). The important factor that may affect the synchronization accuracy is the devices’ internal clock drift, which might be caused by instabilities and noise levels on the devices’ power supply voltage, and temperature variations. For applications where higher synchronization accuracy is desired, it is advisable that clock stabilization techniques should be used as proposed in [38,39].

### 3.4. Visualization of the Wheelchair and User Data

**Figure 13:**
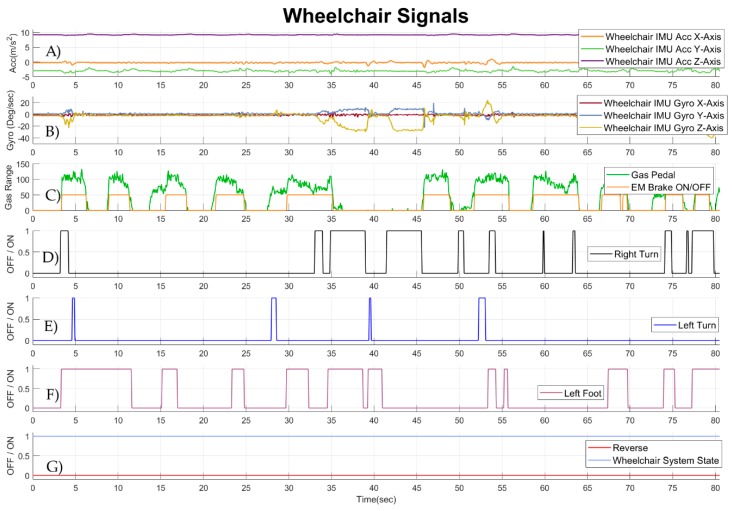
Signals from the wheelchair head-foot steering system of a DCP patient, while he was performing standardized wheelchair start and stop driving task. (**A**) Signals from the accelerometer of the IMU mounted on the wheelchair. (**B**) Signals from the gyroscope of the IMU mounted on the wheelchair. (**C**) Signal from the gas pedal of the wheelchair (green color); and signal from the EM brake of the wheelchair (orange color). (**D**) Signal from the sensor of the wheelchair responsible for right steering (**E**) Signal from the sensor of the wheelchair responsible for left steering (**F**) Signal from the sensor mounted on the left foot support of the wheelchair (**G**) Signal that shows if reverse wheelchair movement is enabled (red color); and signal that shows whether the wheelchair is enabled (blue color).

**Figure 14:**
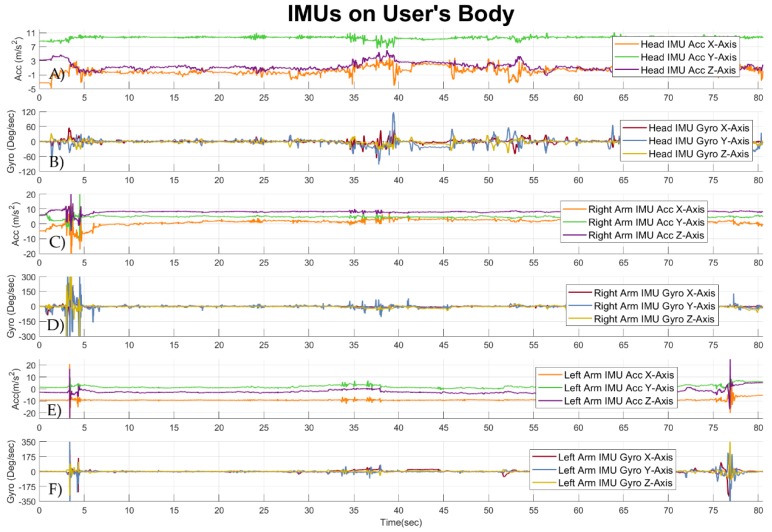
Signals from the five IMUs placed on the body of a DCP patient, while he was performing standardized wheelchair start and stop driving task. (**A**) Signals from the accelerometer of the IMU placed on the forehead of the user. (**B**) Signals from the gyroscope of the IMU placed on the forehead of the user. (**C**) Signals from the accelerometer of the IMU positioned on the right distal arm of the user. (**D**) Signals from the gyroscope of the IMU positioned on the right distal arm of the user. (**E**) Signals from the accelerometer of the IMU attached to the left distal arm of the user. (**F**) Signals from the gyroscope of the IMU attached to the left distal arm of the user.

A full data set that can be captured by the wheelchair data logger and the interface with the IMUs on the body of the user are presented in Figure 13 and Figure 14, respectively. Hereby, a DCP patient was driving the head-foot steering wheelchair. Data from the head-foot steering system of the wheelchair sensors and the IMU mounted on the wheelchair can be seen in Figure 13. Signals from the accelerometer and the gyroscope of the IMU mounted on the wheelchair are presented in Figure 13A,B, respectively. In Figure 13C the amount of gas (green color) and whether the electromagnetic brakes (EM) of the motors are enabled or disabled (orange color) can be observed. The value of 50 for the EM brakes was selected for a clear visualization. Moreover, it can be observed that the brakes get disabled when the gas pedal reaches a certain amount.

Moreover, in Figure 13D,E the steer right and steer left commands of the wheelchair can be seen. For both right and left steering sensor signals, the value of 0 means that the sensor is not pressed, thus the user is not performing any steering command. When these values appear to be equal to 1, the sensor is pressed, and the user steers the wheelchair to the right or to the left accordingly. In Figure 13F the left foot sensor of the wheelchair is presented. A value of 1 means that the sensor is pressed, while a value of 0 means that the sensor is not pressed. This particular sensor was not assigned with any functionality on this wheelchair; however, it was possible to capture the state of the sensor. Finally, the selection between forwards and backwards is presented in Figure 13G (red color). The value of 0 implies that the wheelchair was always in forwards navigation mode. The state of the wheelchair control system with a value of 1 denotes that the wheelchair was always enabled during the driving task.

Due to the fact that the data logging device provides an off-line synchronization interface to connect to multiple IMUs, an arbitrary set of IMUs can be placed on the user. A data set of synchronized signals from the IMUs placed on the body of the DCP patient synchronized in time with the data from the sensors of the wheelchair steering system are presented in Figure 14. The signals of the IMUs were taken from the same DCP patient, while driving the wheelchair. In Figure 14A,B signals from the accelerometer and the gyroscope of the IMU placed on the head of the DCP patient can be seen respectively. Signals from the accelerometer of the IMU positioned on the right distal arm of the user are presented in Figure 14C, while signals from the gyroscope of the IMU positioned on the right distal arm of the user are presented in Figure 14D. In Figure 14E signals from the accelerometer of the IMU attached to the left distal arm of the user are visualized. In Figure 14F signals from the gyroscope of the IMU attached to the left distal arm of the user are shown.

Signals from the IMUs placed on the on the right and left tibialis anterior of the DCP patient are excluded from the figures. During wheelchair driving, the legs of the user were strapped on the foot supports of the wheelchair. Consequently, the IMUs did not capture any artifacts. The action of strapping the legs of the user is a clinical approach that eliminates involuntary movements of the legs. It has been observed that it offers better wheelchair control for the user.

### 3.5. Comparison of Driving Pattern Between DCP Patients and a Healthy Subject

In the first comparison, data were captured by the wheelchair data logger while the five DCP patients were performing a standardized wheelchair driving task. During the task, the DCP patients were asked to perform a 360° turn in place to the right and a 360° turn in place to the left. The driving task was selected to quantify the ability of the DCP patients to control the right and left steering of the wheelchair system. In this task the DCP patients should keep either the right or the left sensor on the head support of the wheelchair enabled for a prolonged time in order to perform the 360° turns. Additionally, a healthy subject was requested to perform the same standardized task using the wheelchair head-foot steering system. Only one healthy subject participated due to the fact that a healthy person, who has full control of body movements, is able to constantly activate either the left or right head support sensor of the wheelchair.

**Figure 15:**
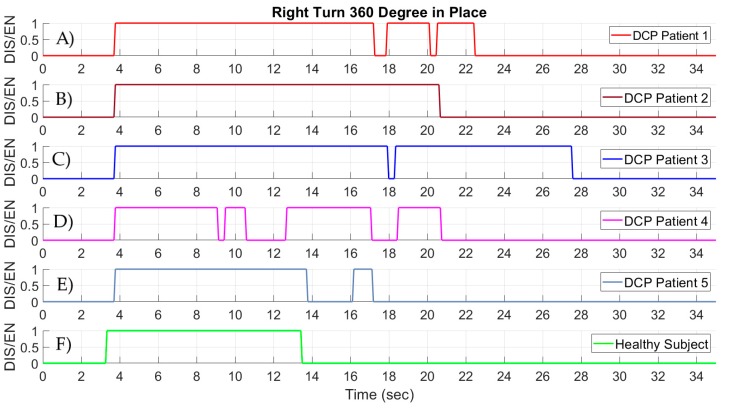
Signals from the head-foot steering system while performing a standardized 360° turn to the right in place. (**A**–**E**) Signals from the sensor responsible for the steer right command of the wheelchair of five DCP patients. (**F**) Signals from the steer right command of the wheelchair of a healthy subject.

The results from the 360° turn in place are presented in Figure 15. The findings from the five DCP users can be observed in Figure 15A–E, while in Figure 15F the ability of the healthy subject used as a guideline can be seen. Initially, it is clearly visible that the required completion time for the 360° right turn in place for the DCP patients is considerably higher compared to the time of the healthy subject. This is due to the fact that DCP patients have less ability to control a wheelchair in higher speed settings than a healthy person. Moreover, it is explicit that four out of five DCP patients need more than one activation on the sensor responsible for the right steering of the wheelchair. Consequently, DCP patients cannot apply constant pressure to the head support, due the involuntary body movements and abnormal postures that are caused in DCP. On the other hand, a healthy subject can easily perform the 360° right turn in place by constantly keeping the right steer sensor enabled without any interrupts.

**Figure 16:**
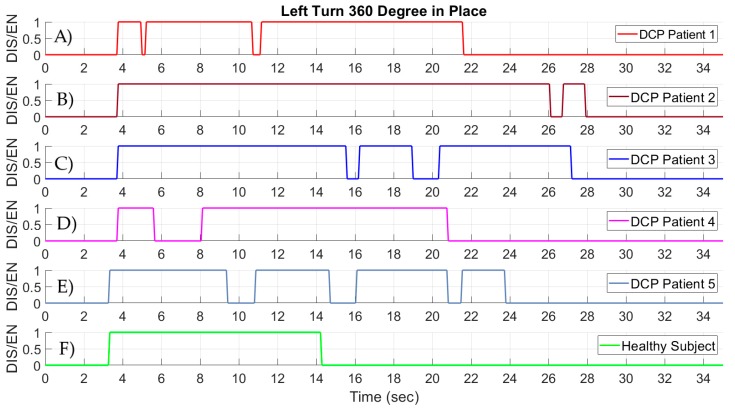
Signals from the head-foot steering system while performing a standardized 360° turn to the left in place. (**A**–**E**) Signals from the steer left command of the wheelchair of five DCP patients. (**F**) Signals from the steer left command of the wheelchair of a healthy subject.

In Figure 16, the results from the 360° turn in place to the left are given. The driving patterns of the five DCP patients are visualized in Figure 16A–E, and the driving pattern of the healthy subject can be seen in Figure 16F. It can be clearly observed that the execution time of the 360° left turn in place is slower for the DCP patients, similar to the higher time required for the right turn, compared with the healthy subject. In the case of the left turn, none of the DCP participants had the ability to execute the task by activating only once the sensor responsible for the left steering of the wheelchair. Moreover, the number of activations required by the DCP patients is not the same for the right steering and the left steering. Consequently, the ability to control right and left steer commands is not the same. The healthy subject was once again able to perform the task with only a constant activation on the sensor.

The second comparison between a DCP patient and a healthy subject was designed to compare the feet control in the head-foot wheelchair system. Both the DCP patient and the healthy subject were asked to perform a second standardized wheelchair driving task. During the task, both participants were instructed to drive the wheelchair from a point A to a point B, and back to point B. Additionally, while performing the driving task, eight intermediate start and stop commands were given to the participants. The total driving distance was set to 20 m in length. This driving task was selected to quantify the ability of the DCP patient to control the gas pedal of the wheelchair, which is placed on the right foot support. Moreover, the behavior of the left foot can be observed, despite the fact that the sensor on the left foot support of the wheelchair was not assigned to any function.

**Figure 17:**
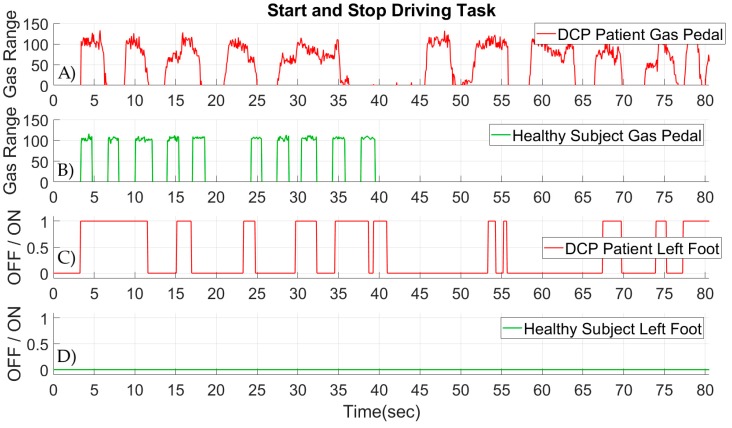
Signals from the head-foot steering system while performing a standardized start and stop driving task. (**A**) Signals from the gas pedal of the wheelchair for a DCP patient. (**B**) Signals from the gas pedal of the wheelchair for a healthy subject. (**C**) Signals from the sensor on the left foot support of the wheelchair for a DCP patient. (**D**) Signals from the sensor on the left foot support of the wheelchair for a healthy subject.

The results from the start and stop driving task are presented in Figure 17. The gas pedal range of the head-foot steering system for a DCP patient is given in Figure 17A, and the gas range for a healthy subject is given in Figure 17B. As observed, the completion time of the task for the DCP patient is twice as high compared to the time of the healthy subject. The major difference between a DCP patient and a healthy subject can be seen in the stability of the gas pedal range. The healthy subject is able to keep the applied pressure constant on the gas pedal of the wheelchair without any variations on the range of the gas pedal. On the other hand, a variability on the range of the gas pedal for the DCP patient can be observed, as the patients lack the ability to keep the applied pressure constant. As far as the behavior of the left foot is concerned, the healthy subject did not apply any pressure to the sensor on the left foot support of the wheelchair, as can be seen in Figure 17C. The DCP patient applied pressure multiple times on the left foot sensor, as presented in Figure 17D. This exhibits the lack of control and coordination of the lower limbs on the DCP patient. The applied pressure on the left foot support is also present when the DCP patient needs to apply pressure on the gas pedal of the wheelchair on the right foot support.

Clear distinctions can be made between a healthy subject and a DCP patient while driving the head-foot wheelchair steering system. The distinctions are with respect to task completion time, number of activations in the wheelchair sensors responsible for the steer right and left command, the ability to stably control the gas pedal of the wheelchair, and lower limb coordination in driving.

## 4. Discussion

In this study, a custom-designed wheelchair data logger for electric-powered wheelchairs used by people diagnosed with DCP is presented that is capable of providing real-time visualization of wheelchair head-foot steering system driving commands. Additionally, the wheelchair data logger provides an interface for off-line signal synchronization with IMUs placed on the body of the user. The proposed data logging system stands out as it is portable and user-friendly. The wheelchair data logger is a battery-powered, handheld device that can easily be carried and is capable of receiving and transmitting data to and from an information resource. Moreover, it is easy to be installed, with a proven installation time of under 5 min. Previous knowledge is not required for the operation of the device. Once it is installed on the wheelchair, its data logging operation starts by the flip of a switch. The installation of the wheelchair data logger is explained in Appendix C.

In contrast to other wheelchair data loggers that have been presented in the literature, our data logger mainly differs in the fact that it captures data from the control mechanism of the wheelchair. Additionally, it provides interfaces to connect to IMUs attached to the patient’s body and offers the option of integrating the measurements from IMUs in the captured dataset. The solution presented here is tailor-made for the DCP target population, since it is one of the most disabling movement disorders and there is not any documented information about the DCP control mechanism. Other systems are based on the installation of sensors on the wheelchair for the purpose of data logging. In [17,18,19] the installation of pressure sensors on the wheelchair seat is required, making it difficult and uncomfortable for the user. In cases such as in [20], alongside the installation of the data logger, modifications in the wheelchair itself needed to be made, where the seat and the armrest of the wheelchair had to be alternated in order to provide housing for the sensors. Moreover, in [21], apart from multiple sensor placement, a netbook had to be mounted on the wheelchair in order to store the acquired data locally. Furthermore, the caster data logger used in [40] for measuring wheel rotation required major modifications, as the wheelchair users were instructed to change the casters of their wheelchairs with the built-in caster data logger.

There are limited studies in the literature that have proposed a similar approach by logging data from the wheelchair control mechanism. In the already existing studies, the approach was to log joystick driving commands. More specifically, a joystick data logger was deployed in [41] with the aim of comparing a conventional movement sensing joystick with an isometric joystick that was developed for people diagnosed with Parkinson’s disease and multiple sclerosis. Another documented attempt to log joystick data from a joystick is proposed in [42], where the authors took a first step in developing an adaptive joystick assistive system for people with movement disorders. It is worth mentioning that a participant diagnosed with CP was also included in this study. Furthermore, joystick data logging was implemented with children diagnosed with CP, by assessing the effectiveness of joystick use and muscle activity while navigating using a joystick [43]. However, the severity of the CP with the participants was not high enough to eliminate the ability to control a joystick. In [44] a data logger for joystick driving commands was utilized to discriminate between beginner and expert control analysis.

Building on the results of this study will have an effect on further studies. The analysis of head-foot steering control signals might constitute an objective tool for the measurement of powered wheelchair driving skills and the assessment of the performance [41,44]. This tool may be useful for the clinical assessment and training of the DCP target population during electric-powered wheelchair driving. Head-foot steering data alongside clinical observation may contribute towards the optimization of training strategies. Offering insights into head-foot control strategies for the early stages of the learning process, could lead DCP users into better and safer driving techniques. What is more, data from a head-foot steering system could provide an effective technique for quantifying key aspects of DCP target population driving skills. In addition, it may make it possible to discriminate between users based on their driving style, so as to customize the training process based on the user.

In addition, the developed data logger is capable of acquiring all signals related to the wheelchair driving control and can also be used as a control device for wheelchair driving training for DCP users in the future. By developing a computer-based environment, which will serve as an exergame for virtual wheelchair driving, the user would have the ability to receive extra indoor training. A middleware could be designed that transmits wheelchair sensor data into the game for serving this purpose. A similar exergame has been proposed to improve physical rehabilitation for people diagnosed with motor function impairments [45]. Related virtual environments and computer games for increasing wheelchair driving via joysticks have been presented in [46,47]. Since in this study, the wheelchair control commands are synchronized in time with IMU data from the user’s body, a further approach could consider the effect of involuntary movements on driving skills. The effect could possibly be seen by quantifying the characteristics of involuntary movements in the DCP target population. Similar quantification has been implemented in the involuntary tremor movement characteristics of patients diagnosed with Parkinson’s disease [48,49,50].

Finally, despite its advantages, the proposed data logger also exhibits a few limitations. To begin with, the proposed data logger is custom-designed only for capturing data from the Adremo head-foot wheelchair steering system, thus cannot find application in other types of electric-powered wheelchairs. Moreover, if there is a future change in the control mechanism of the wheelchair head-foot steering currently used by the DCP population, the data logger might not still be compatible with them. However, due to its reconfigurability and because of the interface between the wheelchair sensors and the MCU board, the system may possibly exhibit the ability to be adapted to changes. The wheelchair data logger might have a considerable large power consumption; nevertheless, it aims to log data for a short period of time. Additionally, the use of common batteries for powering the device can be eliminated by powering up the device directly from the powerful batteries of the wheelchair.

## 5. Conclusions

In this work a portable data logger system was developed with off-the-shelf components. The system is microcontroller-based; thus, it can be easily reprogrammed and provides real-time information of wheelchair head-foot driving. Due to the inability of DCP patients to control the fine movement of the limbs and the presence of involuntary movements, this system can be used for clinical research to gain insights into the mechanism of DCP during electric-powered wheelchair use, adapt the driving training of the user and evaluate driving performance. Wheelchair control commands are synchronized in time with IMU-based data from the body of the user. In the early results, clear distinctions can be made between a healthy subject and a DCP patient, while driving the head-foot wheelchair steering system. The data logging system was successfully used to measure wheelchair mobility behavior of 10 DCP patients during physiotherapeutic driving tasks.

## Figures and Tables

**Figure A3:**
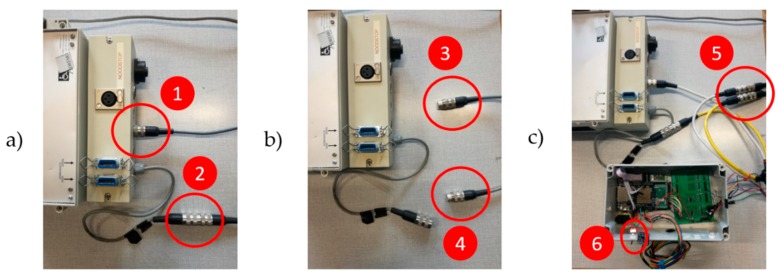
(**a**) **1.** Connector from the wheelchair’s gas pedal on foot support; **2**. connector from the wheelchair’s head support for steering; (**b**) **3 & 4**. unscrew foot support and head support sensors connector; (**c**) **5.** connect the data logger between the sensors and the wheelchair control box; **6.** flip the switch and the data logging starts.

## Abbreviations

The following abbreviations are used in this manuscript:

DCP	Dyskinetic Cerebral Palsy
IMU	Inertial Measurement Unit
GPS	Global Positioning System
CP	Cerebral Palsy
DoF	Degree of Freedom
RTC	Real-Time Clock
GB	Gigabyte
SPI	Serial Peripheral Interface
CS	Chip Select
MISO	Master In Slave Out
MOSI	Master Out Slave In
SCL	Bus Clock Line
I2C	Inter-Integrated Circuit
UART	Universal Asynchronous Receiver-Transmitter
BT	Bluetooth
V	Volt
mA	milliampere
ADC	Analog to Digital Converter
LED	Light Emitting Diode
ECG	Electrocardiogram
s	Standard Deviation

## Appendix A

**Table A1:** List of wheelchair models that were used during the data collection and the data logging device guaranteed to not damage their electronics. All wheelchairs were equipped with the Adremo head-foot steering system; Version 1.3.

Wheelchair Models
Sunrise Medical Hippo
Invacare Moover 785
Sunrise Medical You–Q Alex
Permobil F3 Corpus

## Appendix B

The experimental set up that was used to estimate the power and the current consumption of the wheelchair data logger can be seen in Figure A1. The experimental set up that was used to estimate the transmission latency of the wheelchair data via BT is shown in Figure A2.

## Appendix C

The installation of the data logger is presented in Figure A3. Initially, cables from the wheelchair’s head-foot steering sensors are connected to the wheelchair control electronics. Then, the cables from the wheelchair’s sensors need to be unscrewed for the terminals of the control electronics. The final step is to interface the data logging device by connecting it between the head-foot steering system sensors and control electronics. The data logging starts with the flip of a toggle switch on the data logger itself.
